# Maternal Embryonic Leucine Zipper Kinase (*MELK*) in Cancer: Biological Functions, Therapeutic Potential, and Controversies

**DOI:** 10.3390/biology15020200

**Published:** 2026-01-21

**Authors:** Alaeddin M. Alzeer, Saad Al-Lahham

**Affiliations:** Department of Biomedical Sciences and Basic Clinical Skills, Faculty of Medicine and Allied Medical Sciences, An-Najah National University, Nablus P.O. Box 7, Palestine; s12170254@stu.najah.edu

**Keywords:** *MELK*, CRISPR/Cas9, RNAi, OTSSP167

## Abstract

The Maternal Embryonic Leucine Zipper Kinase (*MELK*) gene is a member of the Snf1/AMPK serine/threonine kinase family. *MELK* has recently attracted considerable interest in cancer biology due to its aberrant overexpression in various malignancies, including glioma, breast, lung, colorectal, gastric, and hematological cancers. It has been shown that higher *MELK* levels are often correlated with unfavorable prognosis, aggressive tumor manifestations, resistance to treatment, and stem-like tumor morphologies. In this review we aim to summarize the current understanding of *MELK* biology, including its functions in cell cycle regulation, apoptosis, oncogenic signaling pathways, and tumor stemness. We also discuss the therapeutic potential, limitations, and controversy of *MELK* inhibitors, and implications in cancer diagnosis and treatment. *MELK* may not be a universal driver oncogene; nonetheless, it is consistently linked to aggressive disease, underscoring its potential as a prognostic biomarker and a candidate for therapeutic co-targeting in combination treatments.

## Simple Summary

*MELK* is a serine/threonine kinase that regulates cell cycle progression, proliferation, apoptosis, migration, and stemness. *MELK* is overexpressed in many solid and hematologic malignancies. Furthermore, high *MELK* levels correlate with aggressive disease, poor survival, treatment resistance, and cancer stem-like features. Several small-molecule inhibitors and RNAi-targeting *MELK* have suppressed the development and progression of various cancers. OTSSP167 has shown promising results in phase I and II clinical trials. However, a study employing CRISPR/Cas9 knockout in breast cancer cell lines reported that loss of *MELK* did not impair proliferation. The review concludes that *MELK* is a robust prognostic biomarker of proliferation and aggressive behavior across multiple cancers but is not a universal driver of tumor growth. Discrepancies may arise from off-target effects of RNAi or inhibitors, context differences across cancer types, and differences in study settings. *MELK* is proposed as a conditional oncogenic vulnerability, especially in p53-deficient triple-negative breast cancer (TNBC) and glioma stem cells. Further research is needed to clarify the target selectivity of inhibitors, context-specific dependencies, and the optimal use of *MELK*-focused agents in combination therapies rather than as standalone treatments.

## 1. Introduction

**Figure 1:**
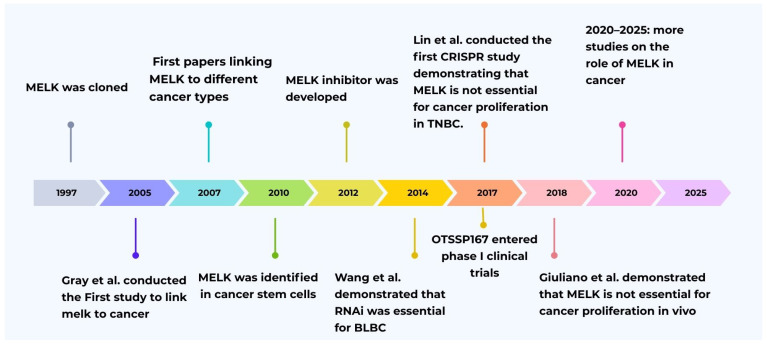
Historical timeline of *MELK* research evolution [1,9,11,12,13,14,15,16,17].

*MELK* gene is a member of the Snf1/AMPK serine/threonine kinase family. *MELK* cDNA was initially cloned in 1997 (Figure 1) and was initially recognized through its expression among several early embryonic cellular stages [1,2]. This initial finding implicated *MELK* in essential developmental processes, including embryogenesis and cell cycle regulation [3]. Additionally, it serves as a multifunctional kinase, participating in various biological and cellular processes. These functions include the stringent regulation of cell cycle progression [4], enhancement of cell proliferation [3], enhancement of mitosis [5], modulation of apoptosis [3,6], promotion of cell migration [7], and support for cell renewal [4]. Furthermore, *MELK* plays a role in cancer cells’ survival, growth, invasion, metastasis, and the regulation of the tumor microenvironment [5]. The extensive body of research has reported elevated *MELK* levels across various cancer types, with *MELK* overexpression frequently correlating with unfavorable prognostic outcomes and promoting tumorigenesis by inhibiting apoptosis [6,7]. Therefore, one can envision that *MELK* knockdown or inhibition may induce apoptosis and disrupt cancer cell proliferation [8]. Indeed, it has been shown that the knockdown of *MELK* enhances the sensitivity of cancer cells to radiotherapy [8,9]. However, few studies have opposed the findings regarding the *MELK* gene’s involvement in cancer [10,11,12]. Therefore, we aim to provide a comprehensive overview of *MELK*’s role in key functions, including proliferation, metastasis, apoptosis, and oncogenesis, as well as the role and therapeutic implications of *MELK* inhibitors. Finally, the contrasting viewpoints concerning *MELK*’s involvement in cancer proliferation and the potential of *MELK* inhibitors to impede cancer cell growth.

## 2. Structure of *MELK*

**Figure 2:**
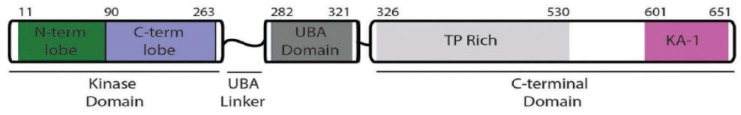
Ref. [25] *MELK* structure: Protein kinases domain is encoded from codon 11 to 263. UBA domain is encoded from codon 282 to 321. TP dipeptide-rich region is encoded from codon 326 to 530 and kinase-associated region 1 (KA1) domain is encoded from codon 601–651 of the C-terminal regulatory region. *MELK*’s phosphorylation activation sites are located at Thr167 and Ser17 [5].

*MELK* gene is located on chromosome 9p13.2. The gene sequence comprises 2501 base pairs and includes 22 exons, encoding 651 amino acids that result in a 70 kDa protein [1,2]. *MELK* is very similar across species, from humans to *C. elegans*, and the crystal structure of a human *MELK* fragment comprises three distinct domains (Figure 2). The N-terminal Ser/Thr kinase domain, ubiquitin-associated (UBA) domain, and C-terminal regulatory region [1,18]. The N-terminal Ser/Thr kinase domain is a catalytic domain with kinase activity that phosphorylates target proteins at their serine or threonine residues [1,19]. The C-terminal domain serves as a regulatory domain, and the structure consists of a segment rich in proline and threonine (TP-rich) and a kinase-associated segment (KA1) [1,18]. The C-terminal KA1 domain has been identified as auto-inhibitory, capable of interacting and controlling the N-terminal of the AMPK-related kinases [1]. Furthermore, it contains a membrane-association domain that binds acidic phospholipids, such as phosphatidylserine, thereby facilitating kinase interactions with substrates or signaling complexes located within the membrane [20,21]. The TP-rich region consists of several phosphorylation sites, with specific Thr phosphorylation in this region being essential for *MELK*’s inhibition of spliceosome assembly. Additionally, the TP-rich domain is phosphorylated in mitotically arrested cells and facilitates binding to the FHA (forkhead-associated) domain of the transcription and splicing factor nuclear inhibitor of protein phosphatase 1 (NIPP1) [1,22]. The Ubiquitin-Associated (UBA) domain of *MELK* is a key regulatory part that is positioned right next to the N-terminal Ser/Thr kinase domain. It is well established that classical UBA domains bind ubiquitin; however, the UBA domain in *MELK* (and other AMPK-related kinases) lacks this property and binds ubiquitin poorly. Instead, its main function is to control the kinase domain’s catalytic activity and make sure it is in the right conformational shape [18,23]. *MELK* is regulated by the UBA domain, which functions as an obligatory structural cofactor. It binds to the back of the kinase N-lobe, stabilizing the kinase C-helix in the active conformation and thereby enabling catalytic activity [20,24]. Additionally, it is shown that eliminating the UBA domain from *MELK* makes the enzyme inactive. This indicates that the UBA domain is not just a regulatory element; it is also essential for *MELK*’s catalytic activity to be expressed [1].

## 3. Role of *MELK* in the Cell Cycle and Apoptosis

*MELK* is expressed in several tissue types via distinct mechanisms [26]. *MELK* expression is observed mainly in proliferative cells like embryonic cells, spermatogonia, and oocytes [13,27]. During these stages, *MELK* expression is tightly regulated, indicating its critical role in determining cell fate and differentiation [28]. *MELK* expression is significantly associated with the cell cycle. Throughout the cell cycle, *MELK* mRNA and protein levels fluctuate. Levels peak during the G2/M phases and subsequently decrease after cell division [9]. Its expression is low in non-growing organs and high in dividing tissues, and it is also linked to well-known proliferation markers, such as MKI67 (a nuclear protein widely used as an indicator of cell proliferation). *MELK* expression is strongly associated with mitotic activity in human cancers [12].

**Figure 3:**
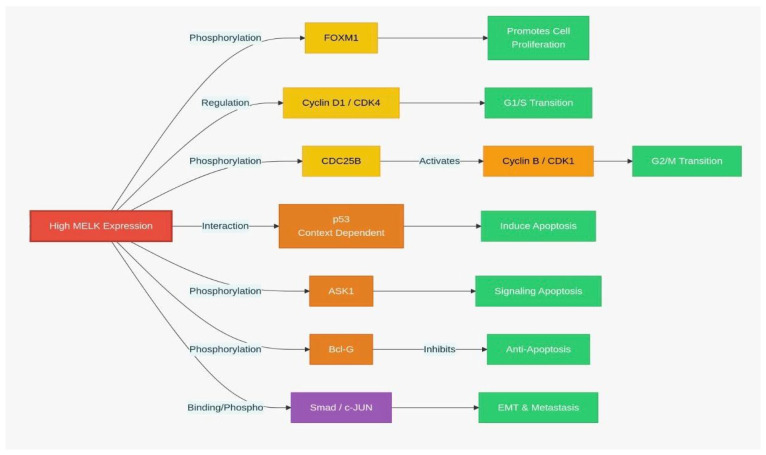
*MELK* signaling pathway: *MELK*-dependent regulation of cell cycle and apoptosis. (EMT: Epithelial–mesenchymal transition.

*MELK* also promote the G1/S transition in the cell cycle [29]. Overexpression of *MELK* has been shown to increase cyclin D1 and CDK4 levels, which are essential for cell transition from G1 to S phase and for initiation of DNA replication (Figure 3) [29,30]. Conversely, inhibiting *MELK* by OTSSP167 prevents cell division and transitions from G1 to S phase by decreasing the levels of cyclin E1 and cyclin D1 (25). Furthermore, *MELK* influences the ATM/CHK2/p53 pathway during the G1/S transition. *MELK* inhibition activates this pathway, leading to cell-cycle arrest at the G1/S phase. Regulation of the *MELK* can activate p53 and p21 downstream, thus effectively inhibiting cyclin-dependent kinase (CDK) activity and transcription factors (E2Fs), which are essential for DNA replication during the G1/S phase [29]. However, the interaction between *MELK* and p53 is complicated. *MELK* has been reported to phosphorylate Ser15 at amino-terminal transactivation domain of p53, thereby inhibiting the G1/S phase transition. This phosphorylation enhances p53 stability and activity, potentially leading to cell cycle arrest and apoptosis [5]. This apparent contradiction suggests that *MELK*’s role in regulating p53 is context-dependent. In certain cancer situations or when cells are under significant stress, *MELK* may activate p53 to induce apoptosis or halt the cell cycle as a protective mechanism. However, in other cases, such as when *MELK* is overexpressed, which frequently occurs in various malignancies, its primary function may shift towards promoting growth and facilitating the G1/S transition. This shift can occur by bypassing p53-mediated checkpoints or by engaging alternative pathways that counteract the effects of p53 [3,5,31]. This illustrates the complexity of *MELK*’s signaling network; the effect of this factor on promoting or inhibiting is an ongoing area of research, with evidence supporting both functions. This demands further investigation into its role in apoptosis.

Additionally, *MELK* is pivotal in activating Fork head Box M1 (*FOXM1*), a critical transcriptional and carcinogenic factor that controls the expression of numerous mitotic transcription factors [3,32]. Expression of *FOXM1* is upregulated in various cancer types, including breast, liver, prostate, brain, lung, colon, and pancreatic cancers [32,33]. The activation of the *MELK*-*FOXM1* axis increases the transcription of Enhancer of zeste homolog 2 (*EZH2*), which in turn inhibits the transcription of differentiation genes, thereby promoting cancer cells in preserving their stem-like properties [34]. Additionally, *MELK* interacts with other critical transcription factors, including *c-JUN* [26], Early region 2 binding factor (E2F1) [35], protein tyrosine phosphatase (CDC25B) [36], zinc finger protein analog (ZPR9), nuclear inhibitor of serine/threonine protein phosphatase-1 (NIPP1), transforming growth factor-β (TGF-β), signal transduction Smad protein, apoptosis signal-regulated kinase 1 (*ASK1*), and pro-apoptotic gene Bcl-G (Figure 3) [37].

## 4. Evidence Supporting the Role of *MELK* in Cancer

The initial investigation regarding *MELK* expression in tumor tissue and its connection to tumor growth was conducted by Daniel et al. in 2005 [5]. High *MELK* expression has been studied in many types of cancer, such as colon, breast, ovary, and lung cancer, compared to normal tissue [5]. This study was followed by numerous other studies over the next two decades, which demonstrated high *MELK* expression in many cancer types, including glioblastoma, breast cancer, prostate cancer, gastric cancer, liver cancer, ovarian cancer, and endometrial cancer (Figure 1) [5,14]. Conversely, *MELK* expression knockdown by siRNA has been shown to inhibit proliferation in cervical, breast, colorectal, pancreatic cancer cells, and other cancer types [13]. This led to the development of *MELK* inhibitors; in 2012, OTSS167 was found to effectively inhibit growth and induce apoptosis across multiple cancer types, including breast cancer, acute myeloid leukemia, and small-cell lung cancer (Figure 1) [15,38]. Moreover, high *MELK* expression is associated with aggressive disease and poor clinical outcomes for different cancer types and correlatively decreased overall survival (OS), recurrence-free survival (RFS), and distant metastasis-free survival (DMFS) (Table 1) [39]. However, the mechanisms by which *MELK* facilitates aggressive tumor growth remain incompletely elucidated [40].

**Figure 4:**
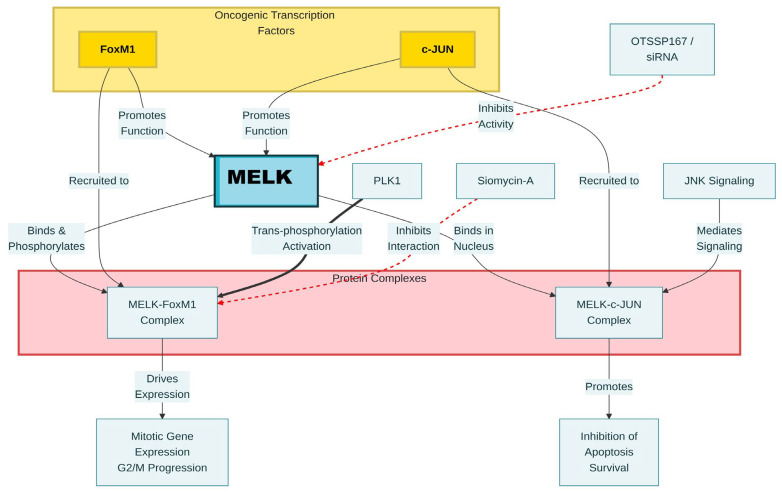
Molecular pathway of *MELK* interactions in glioma stem-like cells. Black arrows indicate activation, and red arrows indicate inhibition/suppression.

Regarding molecular mechanisms, in cancer stem cells, *MELK* function is promoted by other oncogenic transcription factors, such as *FoxM1* and *c-JUN* [16,26]. *MELK* interacts with *FoxM1* in glioma stem-like cells (GSCs), promoting *FOXM1* phosphorylation and activation, thereby increasing the expression of mitotic regulatory genes in GSCs. Furthermore, the activation of *FOXM1* driven by *MELK* is regulated through the binding and subsequent trans-phosphorylation of *FOXM1* by the kinase PLK1 [16]. *MELK* interacts with c-JUN in the nucleus, forming a GSC-specific protein complex that is not observed in normal cells. JNK-mediated *MELK*/c-JUN signaling inhibited apoptosis and enhanced the survival of GSCs. Inhibiting *MELK*-*FOXM1* interaction by treatment with siomycin-A leads to cell cycle arrest in G2/M phase [16]. Furthermore, the GSCs’ sensitivity to radiotherapy can be improved via *MELK* knockdown using siRNA or shRNA, leading to apoptosis of GSCs and a reduction in tumor formation, or treatment with the *MELK* inhibitor (OTSSP167), thereby enhancing their therapeutic response (Figure 4) [4,26,41].

**Figure 5:**
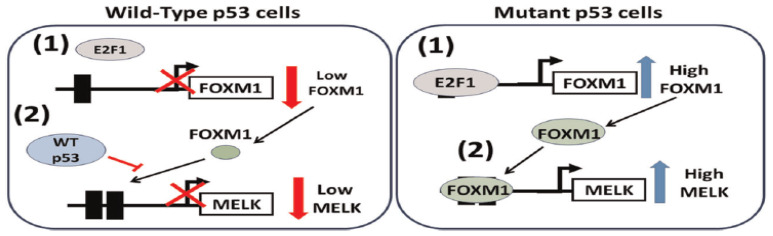
Ref. [42] *MELK* regulation by *FOXM1*, p53, and E2F1A in breast cancer. Wild- type: (1) E2F1 shows decreased binding to the *FOXM1* promoter, (2) p53 decreases *FOXM1* binding for the *MELK* promoter lead to decreased *MELK* expression. Mutant p53: (1) E2F1A induces *FOXM1* expression. (2) *FOXM1* binds with the *FOXM1* binding sites in the *MELK* promoter, thereby increasing *MELK* expression.

In the same context, *MELK*, regulated by E2F1 and *FOXM1* in breast cancer, is contingent upon the mutational status of p53. This mechanism has been explicitly defined, especially in p53-mutant triple-negative breast cancers (TNBCs). E2F1 shows decreased binding to the *FOXM1* promoter in wild-type p53 breast cancer cell lines, thereby lowering *FOXM1* expression and, consequently, reducing *MELK* expression. Conversely, the absence of wild-type p53 activity results in increased *MELK* levels in p53-mutant breast tumors. (Figure 5) [42]. Nevertheless, Mutated p53 failed to inhibit *FOXM1* and *MELK* expression, thereby promoting tumor development in TNBCs. This suggests that mutant p53 is responsible for inducing *MELK* expression, which coincides with the growth of TNBC [9]. Moreover, *MELK* knockdown triggers G2 cell-cycle arrest by reducing cyclin B1 expression, increasing p27 expression, and JNK phosphorylation in TNBC cell lines. Additionally, in non-TNBC cell lines, it induces G1 cell-cycle arrest by decreasing the expression of p21, cyclin B1, and cyclin D1, and by reducing cdc2 phosphorylation. This suggests that the tumorigenesis mechanism of *MELK* in TNBC differs from that in non-TNBC cells. Furthermore, *MELK* knockdown via RNAi preferentially reduced BLBC cell proliferation and induced apoptosis, resulting in smaller cell size and increased radiation sensitivity in TNBC [9,43]. Additionally, *MELK* inhibitors such as OTSSP167 downregulate *FOXM1* expression and its target gene. (Figure 4) [23].

In bladder cancer (BC), *MELK* is markedly overexpressed in BCa cells obtained from patient tissues compared with normal tissues. *MELK* expression continues to increase in tandem with tumor progression and is associated with cell proliferation and migration. BC exhibits a unique characteristic compared with other types. *MELK* suppression leads to cell cycle arrest at the G1/S phase by interacting with the ATM/CHK2/p53 pathway. *MELK* downregulation led to sequential phosphorylation of ATM and CHK2, as well as upregulation of p53 and p21 [29].

In chronic lymphocytic leukemia (CLL), *MELK* is overexpressed in CLL cells and in cell lines (EHEB and MEC1) relative to normal B cells. This overexpression contributes to increased CLL cell survival by decreasing apoptosis and promoting G2/M phase transition [44,45]. In CLL, the findings differed from those of previous studies about the effect of CRISPR/Cas9 on *MELK*. In addition to knockdown via siRNA, *MELK* Knockout mediated by CRISPR/Cas9 enhances apoptosis and suppresses proliferation in CLL cells [45]. A substantial body of research indicates that the overexpression of *MELK* in many cancer types is associated with poor prognosis, cancer cell proliferation, metastasis, and chemo-radiotherapy resistance. Furthermore, *MELK* depletion via RNA interference exhibits antiproliferative effects.

**Table 1:** *MELK* expression, prognostic value, and signal pathway in different cancer types. OS: overall survival, RFS: Recurrence-Free Survival, DFS: Disease-Free Survival.

Cancer Type	*MELK* Expression Level	Prognostic Value	Signaling Pathways	Down Regulation	Key Preclinical Findings
Breast cancer (especially TNBC)	Upregulated; highest in aggressive TNBC subtype [25].	Worse OS. Shorter RFS. Increased distant metastasis. Poor prognosis. Radioresistance [3].	*FOXM1* mTORC signaling E2F targets, linked to loss of WT p53 p27 expression and JNK phosphorylation [9,42,46].	G2/M arrest [9].	Knockdown/OTSSP167 Suppresses growth. Enhances radiotherapy sensitivity; synergy with bortezomib [43].
Glioma	Overexpressed [47].	More aggressive behavior. Poor Prognosis. Radioresistance [47]	*FOXM1*/*EZH2 MELK*/*EZH2* complex *c-JUN*/*MELK*/p53 [4,47]	G2/M arrest [47].	Suppresses {GSC s} more effectively. Enhances radiotherapy response [47].
Lung cancer (NSCLC, LUAD, SCLC)	Overexpressed especially in LUAD [48].	Worse prognosis in LUAD. Negative association with OS [48].	PLK-CDC-CDK1 FAK, *FOXM1*, AKT [5,48].	G2/M arrest [48].	Inhibition disrupts the cell cycle. Induces pyroptosis, reduces invasion/metastasis [49].
Gastric cancer	Frequently Overexpressed in primary GCs [50]	Worse Clinical Outcomes: Associated with lymph node involvement, distant metastasis, depth of invasion [50].	CSF-1/JAK2/STAT3 PI3K/AKT signaling FAK/Paxillin [50,51]	G2/M arrest [50].	Knockdown/OTS167 increases chemosensitivity, decreases proliferation [50,51].
Colorectal cancer	Overexpressed; correlated with tumor stages [52].	Worse Prognosis: invasion, metastasis, radiotherapy, chemotherapy resistance [52]	AKT-FAK/Src Pathway AKT/IKK/P65 signaling axis ERK/IKK/P65 signaling axis [52]	G2/M arrest [52].	Knockdown reduces proliferation, migration, and invasion [52]
Melanoma	Overexpression [35].	Worse prognosis, invasion, metastasis [35].	MAPK pathway PI3K/AKT pathway NFκB transcription [35,53].	G2/M arrest [53].	Knockdown/OTS167/*MELK*-8a inhibit proliferation, survival [35].
Hepatocellular carcinoma	Overexpression [54].	Worse OS and DFS. Correlate with advanced stage and grade [54].	*FOXM1* signaling pathways PI3K/mTOR signaling pathway Akt/mTOR signaling activity [55,56].	G2/M arrest [57].	Knockdown induces apoptosis. OTS167 inhibits growth, synergizes with radiation [58].
Bladder cancer	Overexpression [29].	Worse prognosis, invasion, and metastasis [29].	ATM/CHK2/p53 signaling [29].	G1/S arrest [29].	Knockdown/OTS167 induces G1/S cell cycle arrest [29].
Kidney cancer	Overexpression [59].	Worse prognosis, proliferation, and metastasis [59].	PRAS40/mTORC1 *TOPK* *FOXM1* [60,61].	G2/M arrest [59].	Knockdown/OTS167/OTS514 reduces proliferation, enhances apoptosis [60].
Cervical cancer	Overexpression [62].	Worse prognosis, proliferation, Th1/Th2 imbalance [62]	PD-L1 E2F1 [62,63]	G2/M arrest [62].	*MELK*-8A/OTSSP167 reduces proliferation, enhances apoptosis [62]
Ovarian cancer	Overexpression [64].	Worse prognosis, tumor aggressiveness, progression, decreased survival [64].	*TOPK* TP21 [64].	G2/M arrest [64].	OTSSP167 inhibits growth, increased the sensitivity to carboplatin [5,64].
Endometrial cancer	Overexpression in advanced stages [57].	Worse Prognosis Marker of Aggressive [57,65].	JAK2/STAT3 mTOR signaling E2F1 [57].	G2/M arrest [57].	Knockdown/OTSSP167 inhibits growth, decrease tumor size and weight [57,65].
Prostate cancer	Upregulated in high-grade PC [66].	Worse prognosis enhances the survival of PC cells [66].	TOP2A, AURKB, UBE2C, *CCNB2* interaction between *FOXM1* and CENPE [66,67].	G2/M arrest [68].	OTSSP167 induce apoptosis [66].
Osteosarcoma	Upregulated [69].	Worse prognosis; enhances cell survival, proliferation, and metastasis [69].	PI3K/Akt/mTOR signaling pathway regulates PCNA and MMP9 [69].	G2 and S phases [69].	Knockdown/OTSSP167 reduced proliferation, migration, and invasion. Enhance apoptosis and cell cycle arrest [69].
Chronic lymphocytic leukemia (CLL)	Overexpression [45].	Worse prognosis enhances cell survival by reducing apoptosis and the development of CLL [45].	*FOXM1*-cyclinB1/CDK1 [45].	G2/M arrest [45].	Knockdown/Knockout/OTSSP167 Suppressed proliferation, enhanced apoptosis [44,45].
Acute myeloid leukemia	Overexpression [38].	Worse prognosis Accelerates leukemic blasts proliferation shorter Event-Free Survival (EFS) and OS [38].	*FOXM1*-Cyclin B1 CCNB1 and CDC25B eIF4B/MCL1 [38].	G2/M arrest [38].	Knockdown/OTSSP167 inducing myeloid differentiation and apoptosis [38].
Multiple myeloma	Overexpression [70].	Worse prognosis aggressive disease and drug resistance [70].	*FOXM1* CDK1, CCNB1, CCNB12, PLK1, AURKA and KIF11 AKT signaling pathway and MCL-1 and IRF4 protein expression [70,71,72].	G2/M arrest [70].	Knock-down/OTSSP167 reduced myeloma cell proliferation and survival [70,71].
Diffuse large B-cell lymphoma and mantle cell lymphoma	Overexpression [6].	Worse survival in R-CHOP-treated patients [6].	CDC25B activates CDK1 PI3K/Akt/mTOR [6,73].	G2/M arrest [6].	Knock-down/OTSSP167 enhanced apoptosis [6].

## 5. *MELK* Inhibitors

Chemotherapy has served as the most fundamental and prevalent treatment approach in the fight against cancer in recent decades [5,7]. Recent studies have focused on the *MELK* gene inhibitors using small molecules, such as OTS167 [18], Cyclosporine A [74], MELK 8a, MELK 8b [75], HCTMPPK [74], MELK-T1 [23], and Phillygenin (PHI) [75].

## 6. OTS167 (OTSSP167)

OTSSP167 also known as OTS167 is composed of a “((1,5-naphthyridine core that contains methylketone at the 3-position, trans-4-((dimethylamino)methyl)) cyclohexylamino at the 4-position, and 3,5-dichloro-4-hydroxyphenyl at the 6-position))” [76]. OTSSP167, with an IC50 of 0.41 nM, is a novel inhibitor that specifically targets *MELK*, efficiently suppressing the development and progression of various types of cancer. OTSSP167 has shown promising results in phase I and II clinical trials in AML and breast cancer, making it the only *MELK* inhibitor presently available [37,45]. Additionally, OTSSP167 has the effect of reducing the expression of *FOXM1*, cyclin B1, and CDK1, causing cessation of cell cycle progression and arrest at the G2/M phase [44]. On the other hand, OTSSP167 treatment led to cell cycle arrest at the G2/M phase by upregulation of p21 expression [33]. Alternatively, new evidence suggests that OTSSP167 can inhibit cell proliferation via a non-target-specific mechanism since the tumor was still responsive to the compound even after *MELK* knockout [11]. The ambiguity surrounding the primary therapeutic target of a potent compound poses a significant challenge in drug development, despite its advancement in clinical trials.

Accumulated research conducted over the last decade has demonstrated the impact of OTSSP167 on inhibiting the proliferation of several types of tumor cell lines by targeting *MELK* in a dose-dependent manner, including NSCLC, SCLC [15,49], TNBC [9], colorectal cancer [77], kidney cancer [60], cervical cancer [78], ovarian cancer [65,79], prostate cancer [15], osteosarcoma [69], CLL [45], AML [38], and Multiple myeloma [71]. Additionally, OTS167 demonstrated a substantial in vivo inhibitory effect on tumor growth across various human cancer xenograft mouse models [49], the effect demonstrated in prostate cancer [67], endometrial carcinoma [57], neuroblastoma [17] TNBC, lung cancer, prostate cancer, pancreatic cancer [15], adrenocortical cancer [80], and gastric cancer [50].

Furthermore, OTSSP167 has been combined with other compounds to enhance the inhibitory effect on *MELK*. The combination of OTSSP167 with bortezomib results in more potent inhibition of the proliferation of breast cancer cells [81]. Likewise, the OTSSP167 and cyclin-dependent inhibitor (RGB-286638) combination was used in adrenocortical cancer treatment. This synergism yielded a markedly greater antiproliferative impact, increased caspase-dependent apoptosis, and downregulated *MELK* expression [80]. The research suggests that OTSSP167 is a novel and effective compound, warranting further in vivo testing for cancer treatment and clinical trials.

## 7. Cyclosporine A

Due to its high efficacy, the immunosuppressant medicine cyclosporine A (CsA) has found widespread application in organ transplantation [82]. In addition, CsA demonstrates antineoplastic effects against various cancer types, including prostate cancer [83,84]. In prostate cancer, a critical transcription factor E2F8 promotes cancerous characteristics and influences poor prognosis. Furthermore, the *MELK*-E2F8 signaling axis is integral to the biology of prostate cancer. Additionally, CsA significantly reduced E2F8 expression. Therefore, in cases of prostate cancer, E2F8 is a potential target for therapeutic intervention, and *MELK* is a crucial component in regulating the expression of E2F8. Thus, inhibiting either E2F8 or *MELK* improves the sensitivity of prostate cancer cells to androgen receptor-blocking treatment [83].

## 8. Tetramethyl Pyrazine Chalcone Hybrid-HCTMPPK

Tetramethyl pyrazine (TMP), often known as ligustrazine, is a potent alkaloid monomer with very effective bioactive components. Extensive studies have conclusively shown that TMP and its derivatives have significantly inhibited the growth of various cancer cells, including those in liver cancer [85,86], lung cancer [87], colorectal cancer [88], and breast cancer [89]. HCTMPPK (a derivative of TMP) was subjected to molecular docking with the *MELK*, AURKA, and JUN proteins. The interaction between HCTMPPK and *MELK* genes significantly reduced *MELK* expression and hindered the progression of NSCLC. Although HCTMPPK has been proven to inhibit *MELK* expression, the exact mechanisms involved remain unclear [74]. These studies indicate that HCTMPPK may act as an effective anti-tumor chemotherapeutic agent.

## 9. MELK 8a, 8b

The compound MELK 8a, 8b is alternatively referred to as “((1-methyl-4-[4-[4-[3-(piperidin-4 ylmethoxy) pyridin-4-yl] pyrazol-1-yl] phenyl] piperazine))”, also known as Novartis MELK inhibitor 8a (NVS-MELK8a) and (NVS-MELK8b). The compounds inhibit cell proliferation and cell cycle in MELK-dependent MDA-MB-468 cells [90]. Furthermore, MELK 8a demonstrates high selectivity as a *MELK* inhibitor, representing the first dependable alternative to OTSSP167 for functional studies of *MELK*. Additionally, MELK 8a reduces the viability of and diminishes the growth of TNBC cell lines [91]. In addition, it has high selectivity as a *MELK* inhibitor; this inhibition results in a postponement of the mitotic entrance, which is consistent with a transient G2 arrest state [91]. Furthermore, MELK 8a decreased SQSTM1 phosphorylation and inhibited the NF-κB pathway, consequently suppressing the proliferation of melanoma cells [5,35]. These studies indicate that MELK 8a and 8b inhibit the proliferation of cancer cells with high *MELK* expression; therefore, additional research is necessary to clarify the mechanisms of this inhibition.

## 10. MELK-T1

The compound MELK-T1 is alternatively referred to as “2-methoxy-4-(1H-pyrazol-4-yl)-N-(2,3,4,5-tetrahydro-1H-3-benzazepin-7-yl) benzamide))”. MELK-T1 demonstrates significant and selective inhibition of the *MELK* domain, rapidly decreasing endogenous *MELK* levels by causing it to break down through a process that depends on the proteasome, and inhibiting the growth of MCF-7, a breast adenocarcinoma cell line. On the other hand, MELK-T1 is classified as a type I ATP-mimetic inhibitor, binds to *MELK*, and activates the degradation of the MELK protein by stabilizing the ATP-bound conformation [23]. Furthermore, MELK-T1 affects various mechanisms, significantly inducing p53 phosphorylation, prolonging the upregulation of p21, and downregulating *FOXM1* and its target genes. This is consistent with the previously reported effects of OTSSP167 on AML cell lines [15,23]. The inhibition and depletion of MELK protein through MELK-T1 treatment could allow cancer cells to recognize and respond to DNA damage once again, thereby enhancing tumor sensitivity to radiotherapy and chemotherapy [23]. Ultimately, the dual mechanism of inhibiting enzyme function while reducing its cellular abundance may yield a more effective and lasting therapeutic outcome compared to inhibitors that exclusively target catalytic activity.

## 11. The Controversy: The Crucial Role of *MELK* in Cancer Growth (RNAi vs. CRISPR/Cas9 Methods)

RNA interference (RNAi) and Clustered Regularly Interspaced Short Palindromic Repeats (CRISPR)/CRISPR-associated protein 9 (Cas9) systems are two innovative gene-silencing technologies that have transformed molecular biology research and therapeutic advancement. While both can precisely regulate gene expression, they differ fundamentally in their methods and advantages for specific applications. RNAi is a mechanism for transient, potentially reversible gene knockdown at the post-transcriptional level via mRNA degradation. CRISPR Cas9, on the other hand, is an appropriate technology for more permanent knockouts that involve gene editing directly within DNA [92].

The use of RNAi and small molecule inhibitors demonstrated *MELK*’s crucial role as a vital therapeutic target due to the issue of overexpression in various cancers and its association with severe disease phenotypes, chemotherapy resistance, cancer stem cell renewal, and overall cancer proliferation, resulting in the progression of *MELK* inhibitors to clinical trials. Nonetheless, the definitive role of *MELK* in cancer cell proliferation remains a topic of significant debate within the scientific community, characterized by inconsistent results from various experimental methodologies. The variance can be attributed to the researchers’ utilization of a CRISPR/Cas9 methodology, which completely knocked out *MELK*.

However, few research studies have emerged that contradict the findings of prior investigations. Wang Y. et al. (2014) [9], concluded that the proliferation of BLBC cells requires *MELK* activity, whereas luminal breast cancer cell proliferation does not. In addition, post-*MELK* knockdown via RNAi has shown that Exogenous WT *MELK* expression can rescue the growth effects in both cells and tumors [9,48]. Later, Lin A. et al. (2017) [11] used a different technology, CRISPR/Cas9, to generate frameshift mutations in the *MELK* gene, aiming to investigate the impact of *MELK* knockout on cancerous cell lines (Figure 1). Pooled *MELK*-null, seven triple-negative breast cancer cell lines, were created using gRNAs. These lines were compared with the negative control Rosa26 deletion control cells, and the proliferative activity of *MELK* cell lines, whether they were wild-type or mutant, remained unchanged. Based on these findings, *MELK* expression is not essential for TNBC, and it does promote cancer cell proliferation [11]. These findings prompt us to reconsider whether *MELK* inhibitor OTS167 can prevent cancer cell proliferation. If the *MELK* inhibitors stop tumor cell proliferation and tumor cells are not *MELK*-dependent, then OTS167 is successful in inhibiting tumor cell proliferation or its cellular target genes, other than *MELK*. This may lead us to the conclusion that OTS167’s anti-proliferative actions are not due to its suppression of *MELK* [11]. In a different study in 2017, Huang H et al. [10] used integrated chemical and genetic modifications. Researchers have employed a novel method for protein degradation that utilizes chemical agents, CRISPR, and RNAi to investigate *MELK* as a potential therapeutic target for BBC therapy. Also, slight molecule inhibition, gene deletion, or *MELK* exhaustion under standard culture conditions did not significantly affect cellular proliferation. Both gene editing and pharmaceutical suppression of *MELK* in breast cancer cell lines failed to inhibit cell proliferation in vitro [10]. The study’s discrepancy with previous research prompted researchers to investigate the selection effects of OTSSP167 and the potential off-target consequences of short hairpins targeting *MELK*. Regarding this issue, researchers compared the sensitivity to selective *MELK* inhibitors and OTSSP167 in wild-type (WT) and *MELK* knockout (*MELK*−/−) cells. Their conclusion indicated that after treatment with OTSSP167, there are no differences in cell viability, demonstrating that the effect of OTSSP167 on the viability of MDAMB-468 cells is not attributable to *MELK* inhibition and that its target is different from *MELK*. The findings indicate that under typical culture conditions, suppressing or depleting *MELK* alone has no adverse effect on BBC cell line proliferation [10]. In a 2018 study, Giuliano et al. observed that the overexpression of *MELK* alone was insufficient to induce transformation in immortalized cell lines. Despite findings from CRISPR/Cas9 applications, Zhang et al. reported contradictory results in a 2018 study. *MELK* knockout achieved by CRISPR/Cas9, along with siRNA knockdown, enhances apoptosis and suppresses proliferation in CLL cells [45]. The findings indicate that *MELK* is not essential for cancer cell proliferation, both in vivo and in vitro, and indicate that the immediate absence of *MELK* does not result in any notable impairment of cell viability, proliferation, or drug resistance. Furthermore, the results show that *MELK* knockout cells have not developed mutations that make cells more tolerant to the lack of *MELK* [12,93]. Research utilizing CRISPR technology indicates that *MELK* does not play a significant role in the proliferation of cancer cells and is not a viable therapeutic target. These findings, which show no proliferation after *MELK* knockout with CRISPR, do not conclusively support these claims. Even though these studies have conclusively shown that *MELK* is not essential for the proliferation of cancer cells, no actions have been taken by them to argue against the findings that *MELK* knockdown by RNAi can be rescued by exogenous WT *MELK* expression, indicating that *MELK* is essential for proliferation and that the therapeutic potential of decreasing *MELK* should not be disregarded at this time, especially when used in combination with other chemotherapeutic medications [14].

## 12. Discussion

The broad literature analyzed in this paper has shown that *MELK*, a multifaceted oncogenic kinase, plays a role in cancer biology that has not been fully comprehended over decades of study. The main paradox that the overall analysis elicits is the drastic opposition between the functional research in utilizing RNAi and the small-molecule suppressors on the one hand and the genetic deletion through CRISPR/Cas9 on the other. This contradiction essentially questions our perception of *MELK* as a target of therapy and is an aspect that needs to be interpreted cautiously.

## 13. The Experimental Methodology Paradox

The potential mismatch between knockdown experiments using RNAi and the CRISPR/Cas9 knockout is the most obvious gap in the research of *MELK*. Initial studies by Wang et al. 2014 had convincingly proved that *MELK* activity was required to sustain the growth of BLBC, and exogenous wild-type *MELK* was able to rescue growth-inhibitory phenotypes caused by RNAi [9]. Later CRISPR/Cas9 studies by Lin et al. 2017, Huang et al. 2017 failed to confirm these results, finding that the complete elimination of *MELK* by frameshift mutagenesis in TNBC cell lines had little or no effect on cell proliferation [10,11]. This methodological distinction is not trivial. RNAi approaches generate temporary, partial knockdown with residual *MELK* expression, whereas CRISPR/Cas9 induces complete, permanent knockdown. It is possible to interpret such divergent results in three non-mutually exclusive mechanisms: (1) off-target effects of RNAi hairpins that lead to spurious phenotypes; (2) off-target inhibition of other kinases by *MELK* inhibitors that include OTSSP167; (3) cell line-dependent dependencies or adaptive responses that are not compensated for by *MELK* loss through redundant signaling pathways [14].

There is strong evidence on the off-target hypothesis. Huang et al. have shown that when *MELK*-knockout cells were treated with OTSSP167, the same growth-inhibitory effects were observed as when using wild-type cells, a fact that powerfully suggests the anti-cancer effect of the compound, which appears to be present in other targets besides *MELK* [10]. This observation leads to a radical re-evaluation of whether the reported anti-tumor effects of *MELK* inhibition are due to *MELK*-specific actions or to the polypharmacology of effectors targeting a range of kinases. The clinical implications are high because the progression to the Phase I/II clinical trials of OTSSP167 was based on the premise of an on-target mechanism of action.

## 14. Context-Dependent *MELK* Functions

The literature indicates that *MELK* has a context-dependent role in cancer proliferation and that the status of p53 (mutation or not), and the type of molecular subtype of cancer, significantly determine the role of *MELK* in cancer proliferation. Such as in TNBC, *MELK* overexpression is related to mutant p53, compared with wild-type p53 in luminal breast cancers [9]. The mechanistic basis of this regulatory difference involves E2F1 and *FOXM1*, whose binding dynamics at the *MELK* promoter differ between p53-wild-type and p53-mutant cells. Mutant p53 does not suppress *FOXM1* expression, leading to elevated *MELK* levels that stimulate TNBC tumor growth. On the other hand, in breast cancer with wild-type p53, E2F1-p53-based *FOXM1* suppressions cause decreased EMLK [9,42]. This finding indicates that *MELK* can more likely operate as a conditional vulnerability—a treatment target that is not universally active on all cancers but is most likely to be relevant in most instances of p53-deficiency, where the *MELK*-controlled proliferation pathways are the most common.

Furthermore, the observation that *MELK* knockdown produces differential effects across cancer subtypes further supports this hypothesis. In TNBC and glioma stem cells, *MELK* depletion triggers G2/M phase arrest and apoptosis, whereas in bladder cancer and non-TNBC cell lines, G1/S phase arrest predominates [9,29,43]. These mechanistic differences indicate that *MELK* interacts with different regulatory networks depending on the cellular context. *MELK* in TNBC interacts with the *MELK*-*FOXM1* axis that regulates mitotic transcription factors and fosters stem-like characteristics in a coordinated manner using *EZH2* to suppress differentiation genes [9,43]. In GSCs, *MELK* interacts with *c-JUN* to form a GSC-specific protein complex that enhances JNK-induced signaling and inhibits apoptosis [29]. The specificity of these interactions suggests that *MELK* is not a universal oncogene; instead, it functions as a pathway node that becomes critical in specific malignant contexts in which upstream oncogenic signals converge on *MELK*-dependent effectors.

## 15. *MELK* as a Prognostic Biomarker

Despite remaining contentious that *MELK* is necessary in cancer growth, the increasing body of evidence in several cancer types has shown that there is a strong and consistent relationship between *MELK* overexpression and poor clinical prognosis. There is an association of high *MELK* expression with poor overall survival rates, low recurrence-free survival rates, and high distant metastasis-free survival rates in breast cancer and glioma, as well as lung cancer, gastric cancer, colorectal cancer, ovarian cancer, endometrial cancer, kidney cancer, prostate cancer, and hematologic cancer (Table 1). Such a conclusion made by finding a strong correlation in multiple types of cancer shows that *MELK* is a powerful predictor of malignancy and indicative of proliferation potential. Nonetheless, tumor growth may not be required in every situation. The robust correlation between *MELK* expression and the proliferation marker MKI67 in human tumors indicates that *MELK* is an indirect marker of mitotic activity rather than a specific, context-dependent marker of *MELK* activity [12]. Additionally, it appears that *MELK* prognostic value is high, especially in aggressive forms, including TNBC, high-grade glioma, and SCLC, where *MELK* overexpression is linked with poor prognosis and predicts higher aggressiveness. The clinical implications of such a difference are that a biomarker that has a high probability of predicting aggressive disease and poor prognosis has a high clinical value in risk stratification, treatment planning, and patient monitoring, whether it is considered as a direct therapeutic activity or not.

## 16. Therapeutic Implications and Combination Strategies

The literature on the *MELK* inhibitor, mainly focusing on the research of OTSSP167, demonstrates the potential and challenges in the development of the *MELK*-targeted therapy. Pre-clinical experiments have always able to prove that OTSSP167 inhibits tumor cell proliferation, increases vulnerability to chemotherapy and radiotherapy and prevents metastatic capacity in a variety of xenograft models [49]. These results justify further studies of *MELK* inhibition in clinical settings, especially when used as a part of combination therapy. However, the evidence for off-target mechanisms underlying OTSSP167’s anti-tumor activity necessitates a paradigm shift in how these compounds are evaluated.

## 17. Conclusions

Maternal Embryonic Leucine Zipper Kinase (*MELK*) is also considered as an important oncogenic protein, and it is overexpressed in most human malignancies. *MELK* overexpression can be associated with poor prognosis, treatment aggressive manifestation, treatment re-resistance, and stem-like morphologies of tumors. The correlations reached are evidence that *MELK* is a clinical biomarker of tumor proliferation and malignancy. Despite these observations, the fundamental contradiction between RNAi-mediated studies demonstrating *MELK* essentiality and CRISPR/Cas9 knockout studies showing *MELK* independence cannot be reconciled without acknowledging the probability of substantial off-target effects in both experimental methodologies and therapeutic inhibitors. This unresolved paradox mandates a more nuanced interpretation of *MELK*’s role in cancer biology and necessitates reformulation of therapeutic strategies. Moreover, the anti-tumor activity of OTSSP167 appears to involve off-target mechanisms, further complicating the interpretation of *MELK*’s role as a direct therapeutic driver.

The following conclusions are supported by the evidence provided in the present paper: First, *MELK* is an effective and clinically relevant prognostic biomarker of tumor aggressiveness, proliferative ability and poor clinical outcome across many different cancer types. Second, *MELK* is a conditional oncogenic vulnerability that is most pertinent in particular molecular contexts, specifically, p53-deficient TNBC and glioma stem cells, rather than an all-encompassing oncogenic driver. Third, the antitumor effects of OTSSP167 and related off-target kinase-inhibitory effects probably contribute significantly to antitumor activity and make it challenging to interpret published preclinical data. Finally, *MELK* inhibition may retain therapeutic utility when combined with chemotherapy, radiotherapy, immunotherapy, or multikinase inhibitors, despite uncertainty regarding on-target selectivity.

In the future, the *MELK* field requires several critical research methodologies and improvements in clinical strategies. To determine the on-target mechanisms, first, complete kinase selectivity profiling of all *MELK* inhibitors should be conducted using biochemical assays and cellular studies in isogenic wild-type and knockout backgrounds. Second, the exploration of the *MELK* activities in the tumor microenvironment, specifically, influence on macrophage polarization, immune cell infiltration, and regulation of checkpoint pathways, is an untapped prospect in the context of deepening the knowledge of *MELK*-mediated immunosuppression. Third, mechanistic experiments should determine which molecular subtypes and genetic backgrounds in which *MELK* is a veritable dependency, using patient-derived xenografts, cellular adaptation, and redundancy conclusion primary tumors, and engineered cell lines. Lastly, *MELK*-targeted agents should be developed for clinical use to prioritize combination therapy with existing agents rather than demonstrating efficacy in monotherapy, given their emerging non-essential status in most settings.

Although the functionality of *MELK* as a marker of cancer proliferation remains controversial, its persistent correlation with malignant phenotypes, resistance to treatment, and poor clinical outcomes makes *MELK* a significant biological aspect of aggressive cancers that require further studies and continued investigation. *MELK* may not meet the requirements of a classical oncogenic driver—a solitary protein the inhibition of which is enough to inhibit tumor development—but is an informative biomarker and a promising target in therapeutic co-targeting in multimodal treatment approaches that target multiple dependencies of cancer cells. Future studies should be able to differentiate the utility of *MELK* as a prognostic factor and a therapeutic target by defining context-dependent treatment modalities that may be applied using the biology of *MELK*, but without overgeneralizing its main and context-specific roles in cancer pathogenesis.

## Data Availability

Data sharing does not apply to this article, as no datasets were generated or analyzed during the current study.

## Abbreviations

AFP	Alpha-fetoprotein
AML	Acute myeloid leukemia
*ASK1*	Apoptosis signal-regulating kinase 1
AURKB	Aurora kinase B
BCa	Bladder cancer
BLBC	Basal-like breast cancer
*CCNB2*	Cyclin B2
CLL	Chronic lymphocytic leukemia
CRC	Colorectal cancer
CsA	Cyclosporine A
DLBCL	Diffuse large B cell lymphoma
EC	Endometrial cancer
*ERF1*	Early region 2 binding factor
EMT	Epithelial-mesenchymal transition
*EZH2*	Enhancer of zeste homolog 2
*FAK1*	Focal adhesion kinase 1
*FOXM1*	Forkhead box M1
GBM	Glioblastoma
GC	Gastric cancer
GEO	Gene Expression Omnibus
GSCs	Glioma stem cells
HCC	Hepatocellular carcinoma
HGSOC	High-grade serous ovarian cancer
IHC	Immunohistochemistry
KA1	Kinase-associated segment
LUAD	Lung adenocarcinoma
LULC	Large cell lung carcinoma
LUSC	Squamous cell lung carcinoma
MCL	Mantle cell lymphoma
*MELK*	Maternal embryonic leucine zipper kinase
MIBC	Muscle-invasive bladder cancer
MM	Multiple myeloma
MMP9	Matrix metalloproteinase-9
NHLs	Non-Hodgkin lymphomas
NMIBC	Non-muscle-invasive bladder cancer
NIPP1	Nuclear inhibitor of serine/threonine protein phosphatase-1
NSCLC	Non-small cell lung cancer
OS	Osteosarcoma
PC	Prostate cancer
PCNA	Proliferating cell nuclear antigen
PCL	Plasma cell leukemia
SCLC	Small cell lung cancer
shRNA	Short hairpin RNA
SOC	Serous ovarian cancer
SQSTM1	Sequestosome 1
TCGA	Cancer Genome Atlas
TGF-β	Transforming growth factor-β
TMP	Tetramethyl pyrazine
TNBC	Triple-negative breast cancer
TOP2A	Topoisomerase II alpha
*TOPK*	T-LAK cell-originated protein kinase
UBE2C	Ubiquitin-conjugating enzyme E2 C
ULM	Uterine leiomyoma
ULMS	Uterine leiomyosarcoma
ZPR9	Zinc finger protein analog
